# Bacterial Community Associated with the Intestinal Tract of Chinese Mitten Crab (*Eriocheir sinensis*) Farmed in Lake Tai, China

**DOI:** 10.1371/journal.pone.0123990

**Published:** 2015-04-13

**Authors:** Xiaobing Chen, Panpan Di, Hongming Wang, Bailin Li, Yingjie Pan, Shuling Yan, Yongjie Wang

**Affiliations:** 1 Laboratory of Quality and Safety Risk Assessment for Aquatic Products on Storage & Preservation, Ministry of Agriculture, Shanghai, China; 2 Shanghai Engineering Research Center of Aquatic-Product Processing & Preservation, Shanghai, China; 3 College of Food Science and Technology, Shanghai Ocean University, Shanghai, China; 4 Institute of Biochemistry and Molecular Cell Biology, University of Goettingen, Goettingen, Germany; Fish Vet Group, THAILAND

## Abstract

Chinese mitten crab (CMC, *Eriocheir sinensis*) is an economically valuable species in South-East Asia that has been widely farmed in China. Characterization of the intestinal bacterial diversity of CMC will provide insights into the aquaculturing of CMCs. Based on the analysis of cloned 16S rRNA genes from culture-independent CMC gut bacteria, 124 out of 128 different clones reveal ＞95% nucleotide similarity to the species belonging to the four phyla of *Tenericutes*, *Bacteroidetes*, *Firmicutes* and *Proteobacteria*; one clone shows 91% sequence similarity to the member of TM7 (a candidate phylum without cultured representatives). Fluorescent in situ hybridization also reveals the abundance of *Bacteroidetes* in crab intestine. Electron micrographs show that spherical and filamentous bacteria are closely associated with the microvillus brush border of the midgut epithelium and are often inserted into the space between the microvilli using a stalk-like cell appendage. In contrast, the predominant rod-shaped bacteria in the hindgut are tightly attached to the epithelium surface by an unusual pili-like structure. Both 16S rRNA gene denaturing gel gradient electrophoresis and metagenome library indicate that the CMC *Mollicutes* group 2 appears to be present in both the midgut and hindgut with no significant difference in abundance. The CMC *Mollicutes* group 1, however, was found mostly in the midgut of CMCs. The CMC gut *Mollicutes* phylotypes appear to be most closely related to *Mollicutes* symbionts detected in the gut of isopods (Crustacea: Isopoda). Overall, the results suggest that CMCs harbor diverse, novel and specific gut bacteria, which are likely to live in close relationships with the CMC host.

## Introduction

Chinese mitten crab (CMC, *Eriocheir sinensis*) is an economically valuable species cultured in South-East Asia. In China, aquaculture of this species has increased rapidly since the 1990s. By 2012, the annual output of CMCs has reached approximately 714,380 metric tons, which is more than a 40-fold increase over the production in 1991 [1]. With the rapid growth of intensive cultivation of CMCs, however, various diseases appear and are heavily affecting the sustainable development of the crab industry. For example, trembling disease is one of the most common and severe diseases found in CMCs, which causes high mortalities and heavy economic losses [2]. Accordingly, considerable interest and effort have been put on developing ways to maintain a healthy and reliable aquatic farming environment for CMCs.

The interaction between intestinal bacteria and their hosts has been recognized as a crucial factor affecting the wellness of aquatic animals [3–6]. The gut-associated microbiota play a unique role in the host’s gastrointestinal tract development, nutritional state, immune responses, and disease resistance. The imbalance of host-microbiota homeostasis has been shown to be responsible for certain illnesses [7, 8]. In 2007, Li and coauthors reported that *Proteobacteria* and *Bacteroidetes* were the two dominant bacterial phylotypes found in the intestines of both pond-raised and wild CMCs, with higher inter-subject variation presented in the gut bacterial community of pond-raised CMCs. A significant portion of the phylotypes found in the CMCs did not have close relatives in GenBank database [9].

The goal of this study is to understand the diversity of the intestinal microbiota presents in distinctly farmed CMC populations as well as to shed light on specific intestinal bacteria, which are potential probiotics in CMCs, by using culture-independent 16S rRNA gene amplification and sequencing (e.g., denaturing gradient gel electrophoresis (DGGE), clone library, metagenome library), in situ hybridization with specific fluorescence-labeled oligonucleotide probes (FISH), and electron microscopy. The results presented in this study provide novel insights into the unusual diversity of the intestinal bacteria in CMCs that will be useful for manipulating them with probiotics.

## Materials and Methods

### CMC samples

A total of 18 healthy female crabs (Table 1), which were of higher economic values than male ones, were collected with permission from an aquaculture farm (Fengshou Crabs Farm Food Co., Ltd) in Lake Tai, China (31°13′N, 120°58′E) in November 2012. Crabs in this farm were feed with formula diets containing fish meal, soyabean extracts, fish oil, wheat flour and other microelement (Xinxin #1 and #2 diets, Zhejiang Xinxin Feed Co., Ltd., Jiaxing, Zhejiang Province, China), and were maintained in pond water with pH 7.0–9.0, DO > 3 mg/L, ammonia < 0.4 mg/L and nitrite < 0.15 mg/L. In the laboratory, after crab samples were starved for one week, their body surfaces were washed thoroughly using sterile water and disinfected with 75% ethanol for 2 min. Crabs were dissected immediately after washing. Their digestive tracts were aseptically removed and stored at -20°C for subsequent analysis.

**Table 1:** Crabs used in this study.

Sample ID^1^	Experimental group
A1, A2	Clone library
B1, B2, B3, B5, B6, B7, DM5, DH5, DM6, DH6, DM7, DH7	Fluorescent in situ hybridization
B4, C1, C2	Electron microscope observation
DM2, DH2, DM3, DH3, DM4, DH4	DGGE
DM3, DH3, DM4, DH4	Metagenomic sequencing

^1^ DMx and DHx mean the midgut and hindgut from one crab individual if x is of the same number.

### Culture-independent cloning and sequencing of 16S rRNA gene

Total genomic DNA was extracted from the intestines of two crabs according to the instructions provided by the DNeasy Blood and Tissue Kit (Qiagen, Hilden, Germany). The intestinal bacterial 16S rRNA genes were amplified using universal primer pair 27F (5′AGAGTTTGATCCTGGCTCAG3′) and 1492R (5′GGTTACCTTGTTACGACTT3′) [10]. The PCR reaction contained 2.5 μl of 10× PCR buffer, 0.2 mM dNTP mix, 0.4 μM of each primer, 1.5 U *Taq* polymerase (TakaRa, China), and approximately 100 ng of DNA template. The PCR program was as follows: 95°C for 4 min, followed by 35 cycles of 94°C for 45 s, 55°C for 45 s and 72°C for 1 min, and a single final extension step of 72°C for 10 min. Amplified 16S rRNA gene fragments were purified using the PCR Fragment Purification Kit (Tiangen, Beijing, China) and cloned using the pGM-T Cloning Kit (Tiangen, Beijing, China). The ligated plasmid was transferred into competent *Escherichia coli* T0P10 cells following the manufacturer’s instructions. Inserts of the expected size (approximately 1,500 bp) were confirmed by colony PCR and screened based on restriction fragment length polymorphisms (RFLP) grouping as previously described [11]. Representative clones were sequenced on both strands using primers T7 and SP6 (Mapbioo, Shanghai, China).

The archaeal 16S rRNA gene was amplified using the primer pair 340F (5′CCCTAYGGGGYGCASCAG3′) and 1000R (5′GGCCATGCACYWCYTCTC3′) [12]. The *Archaea* strain (*Haloferax volcanii*) ordered from the Institute of Microbiology, the Chinese Academy of Sciences, was used as positive control. The optimized PCR reaction contained 5 μl of 10× PCR buffer, 2 mM MgCl_2_, 0.5 μM of each primer, 0.16 mM of each dNTP, and 1.25 U *Taq* polymerase (Fermentas, MBI). The PCR program used was as previously described [12].

### Fluorescent in situ hybridization

The intestines of three crabs were homogenized individually in 2 ml of 0.01 M PBS using sterile mortars. The homogenate was centrifuged at 3,220 ×*g* for 2 min. One milliliter of the supernatant was filtered through polycarbonate filters of 0.22 μm pore size (Isopore Membrane Filters, Merck Millipore Ltd). Bacteria remained on the filters were fixed, stored, and hybridized as described in [13] with slight modification. The dehydrated samples were double hybridized with two probes labeled with different fluorophores for 3 h at 46°C and washed for 15 min at 48°C. After stained with Vectashield-DAPI (Vector Laboratories, Burlingame, USA.), the samples were analyzed under a Zeiss Axiophot epifluorescence microscope with filter sets for DAPI, Cy3, and FITC.

The sequences and specificities of the following probes: Eub338 (targeting most of the Eubacteria), EPSY549 (targeting *Epsilonproteobacteria*), CF319a (targeting *Bacteroidetes*), and Eub338 antisence (negative control), were obtained from Probebase (http://131.130.66.201/probebase/) (Table 2).

**Table 2:** Fluorescent probes used in this study.

Probe	Target	Sequence (5’￫3’, Position of *E*. *coli*)	Fluorescein (5’)	Formamide (%)
EUB338	Bacteria	GCTGCCTCCCGTAGGAGT (338–355)	Cy3	20
NONEUB		ACTCCTACGGGAGGCAGC	FITC	20
EPSY549	*Epsilonproteobacteria*	CAGTGATTCCGAGTAACG (549–566)	FITC	20
CF319a	*Bacteroidetes*	TGGTCCGTGTCTCAGTAC (319–336)	FITC	20

### Electron microscopy

Three crab intestines were exposed to and rinsed in 2.5% cold glutaraldehyde solution for 30 min, subsequently removed and immediately stored in 2.5% glutaraldehyde solution for 16 h at 4°C. After two washes in 0.1 M PBS for 15 min each, the fixed samples were washed three times in 0.1 M PBS for 15 min each.

For scanning electron microscopy (SEM), after washing in 30, 50, 70, 90 and 100% of serial ethanol (each for 15 min) at room temperature, the intestines were desiccated in isoamyl acetate for two times (each for 15 min), packed with filter papers and dried in a critical-point dryer (HCP-2 HITACHI, Japan) for 5 h. The dried samples were displayed on a specimen stub with double-sided adhesive tape, coated with gold using a sputter coater (Cressingtom 108 auto) and observed under a scanning electron microscope (S-4800 HITACHI, Japan).

For transmission electron microscopy (TEM), the intestines were serially dehydrated in 30, 50, 70 and 90% of ethanol, and 90 and 100% of acetone (each for 15 min). After soaking in Epon 812 (acetone: resin, 1:1) for 3 h and 100% resin for 2 h, the samples were embedded successively at 35°C for 16 h, 45°C for 24 h and 60°C for 48 h. After air-cooling, ultrathin sections (40 nm) were cut with an ultramicrotome (UC6 LEICA, Germany) and mounted on copper grids, which were contrasted with 2.5% uranyl acetate and lead citrate, and analyzed under a transmission electron microscope (H7700 HITACHI, Japan).

### DGGE analysis of bacterial V1-V3 regions

Total DNA was extracted from the midgut and the hindgut of three crabs, respectively, according to the instructions provided by the DNeasy Blood and Tissue Kit (Qiagen, Hilden, Germany). The V1-V3 regions of bacterial 16S rRNA gene were amplified using the primer pair GC-27F (5′CGCCCGCCGCGCGCGGCGGGCGGGGCGGGGGCACGGGGGGAGRGTTYGATYMTGGCTCAG3′) [14] and 534R (5′ATTACCGCGGCTGCTGG3′) [15]. DGGE was performed under the conditions of a linear 40 to 60% denaturant gradient (100% denaturant contained 7 M urea and 40% deionized formamide), a temperature of 60°C, and a constant voltage of 60 V for 16 h (Bio-Rad, USA). The representative gel bands were excised, mashed with pipette tips and then rinsed in 20 μl of 1× TE buffer at 4°C overnight. The supernatant at 1:10 dilution was used as template for PCR using primer pair 27F (5′AGRGTTYGATYMTGGCTCAG3′) and 534R (5′ATTACCGCGGCTGCTGG3′). The amplified products were purified using the QIAquick Gel Extraction Kit (Qiagen, Hilden, Germany). The purified products were ligated into pUCm-T vector (Sangon Biotech, Shanghai, China) and transformed into *E*. *coli* Top10 cells. Confirmation of inserted fragments (approximately 500 bp) was performed using colony PCR. Three clones from each PCR band were sequenced using primers M13-20 (Sangon Biotech, Shanghai, China).

### Metagenomic sequencing of 16S rRNA gene

Total DNA was extracted from the midgut and the hindgut of two crabs, respectively, as described above. PCR amplification of the V1–V2 variable region (approximately 349 bp) of the 16S rRNA gene was performed using the universal primers 27F (5′AGAGTTTGATCCTGGCTCAG3′) and 356R (5′TGCTGCCTCCCGTAGGAGT3′) [16]. The optimized PCR reaction contained 2.5 μl of 10× PCR buffer, 5 mM dNTP mix, 10 μM of each primer, 0.1 μl of *Taq* polymerase (5 U/μl, TakaRa). Cycle conditions were as follows: an initial denaturation step at 94°C for 3 min, followed by 20 cycles of 94°C for 30 s, 50°C for 30 s and 72°C for 30 s, and ended with a final extension step at 72°C for 5 min. Paired-end multiplex sequencing of the 16S rRNA genes was performed using an Illumina MiSeq instrument (Personalbio, Shanghai, China).

### Clone library and DGGE sequence analysis

All sequences were checked for chimera based on the DECIPHER's Find Chimeras web tool (http://decipher.cee.wisc.edu/FindChimeras.html). 16S rRNA gene sequences without chimera were analyzed using Classifier of Ribosomal Database Project (RDP) with a 95% confidence threshold (http://rdp.cme.msu.edu/classifier/classifier.jsp). Representative sequences were compared to those in the GenBank database using the BLAST program (http://blast.ncbi.nlm.nih.gov/Blast.cgi?). The closely related sequences were downloaded and aligned with the cloned sequences. Phylogenetic trees were constructed using the maximum-likelihood, maximum-parsimony, and neighbor-joining algorithms using MEGA6 (http://www.megasoftware.net/). One hundred replicates were performed for the bootstrap analysis. Based on RDP and phylogenetic analyses, the sequences clustered together were considered to belong to a single phylotype (operation taxonomic unit (OTU)). Local BLAST was performed by Bioedit (http://www.mbio.ncsu.edu/bioedit/bioedit.html) in order to analyze the similarity shared between sequences obtained in this study.

Representation and diversity of the clone library were evaluated with indices of Shannon index, Simpson and Chao-1. Good’s coverage was calculated to determine sequencing depth.

### Metagenomic data analysis

Mothur v.1.11.0 (http://www.mothur.org/) was used to filter low quality reads in raw Illumina fastq data sets. A window with a length of 50 bp was moved through each sequence. Sequences were truncated at the end of the window when the average quality score was less than the threshold of 30 [17]. All clean reads were overlapped by software FLASH (Fast Length Adjustment of Short reads, http://ccb.jhu.edu/software/FLASH/). Reads were truncated at any sites containing more than six consecutive bases, and any reads containing one or more ambiguous base calls were discarded. OTUs were assigned using QIIME’s uclust-based open-reference OTU-picking workflow with a threshold of 97% pairwise identity, and OTUs with more than 0.005% of abundance [18] were clustered at 97% identity against SILVA database (SSU) [19]. Taxonomic assignment from phylum to species level was based on BLAST hits. Sequencing depth was estimated using rarefaction analysis. The Simpson index, Chao 1, Shannon and good’s coverage were calculated using Mothur in order to estimate the genera richness and α-diversity in every sample [20]. Significant taxonomic differences between samples were also calculated by Mothur.

### Nucleotide sequence accession numbers

The nucleotide sequence accession numbers for the 16S rRNA genes cloned in this study are HG792175-HG792251 (clone library) and KM406330-KM406379 (DGGE). The metagenomic sequences were deposited at the NCBI Sequence Read Archive (SRA) under accession numbers SRR1560717-SRR1560720.

## Results

### Gut microbiota composition based on clone library

**Fig 1:**
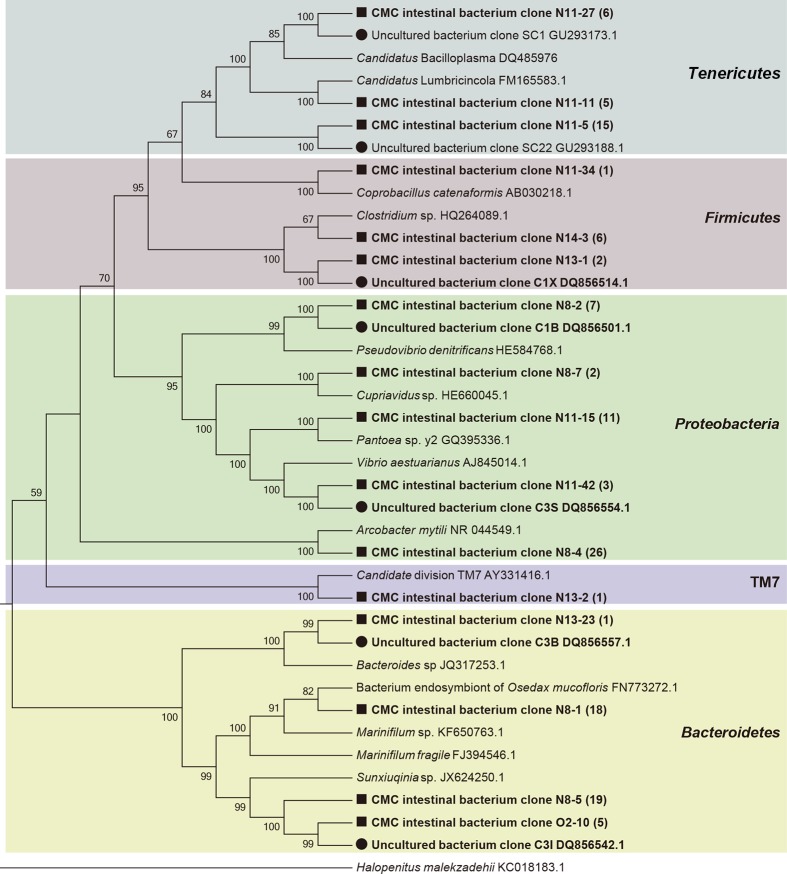
Phylogenetic affiliations of the bacterial 16S rRNA genes cloned from crab intestines. The clones obtained from crab intestines are shown in bold. The clones obtained in this study and those from a previous study (Li et al, 2007) are indicated with black square and black dot, respectively. The number of clones is shown in parenthesis. The background colors indicate the bacterial phylotypes of different phyla.

A total of 222 clones containing nearly full-length 16S rRNA gene were obtained. Based on RFLP screening and grouping, 128 representative clones with distinct RFLP patterns were subjected to sequencing. BLAST sequence similarity and phylogenetic analysis revealed that these 128 clones could be divided into five phylum groups, *Proteobacteria* (38.3%, 49/128), *Bacteroidetes* (33.6%, 43/128), *Tenericutes* (20.3%, 26/128), *Firmicutes* (7.0%, 9/128), and TM7 (0.8%, 1/128) (Fig 1). Based on the percentage of clones in each phylum groups, it indicated that *Proteobacteria*, *Bacteroidetes* and *Tenericutes* were the three dominant phylotypes of the intestinal bacteria in the CMCs collected from Lake Tai. The *Proteobacteria* phylotype, in particular, comprises four bacterial classes, *Epsilonproteobacteria* (26 clones), *Gammaproteobacteria* (14 clones), *Alphaproteobacteria* (seven clones), and *Betaproteobacteria* (two clones) (Fig 1).

At the genus level, these 128 representative clones were divided into 14 OTUs, which comprise eight known genera and six uncultured lineages (Fig 1). For the known genera, the two most abundant phylotypes were closely related to members of *Arcobacter mytili* (ε-*Proteobacteria*) and *Marinifilum fragile* (*Bacteroidetes*), respectively (Fig 1). The second most abundant phylotypes were affiliated with *Pantoea* sp. (γ-*Proteobacteria*) and *Pseudovibrio denitrificans* (α-*Proteobacteria*), respectively (Fig 1). The rest of the phylotypes were related to *Coprobacillus catenaformis*, *Vibrio aestuarianus*, *Cupriavidus* sp. and *Bacteroides* sp. All the sequences in the six uncultured lineages displayed less than 95% identity to known genera in public databases. According to the phylogenetic analysis, one dominant phylotype was closely related to *Sunxiuqinia* sp. (*Bacteroidetes*); three dominant phylotypes had less than 86% identity to the known *Mollicutes* sequences in public databases. Of them, one phylotype (clone N11-11) was closely related to *Candidatus* Lumbricincola, and another phylotype (N11-27) was closely related to *Candidatus* Bacilloplasma (Fig 1).

The coverage of the clone library was at approximately 97.8%, which indicated that the analyzed clones adequately represented the bacterial composition in crab intestines. The Shannon index, Simpson index and Chao-1 of the clone library were 2.229, 0.8714 and 16, respectively, suggesting that the diversity of the intestinal bacteria harbored in CMCs was relative low. No archaeal 16S rRNA gene sequences were amplified, which implied that archaea may be rare or non-existing in the CMC intestines.

### FISH analysis of two dominant phylotypes

**Fig 2:**
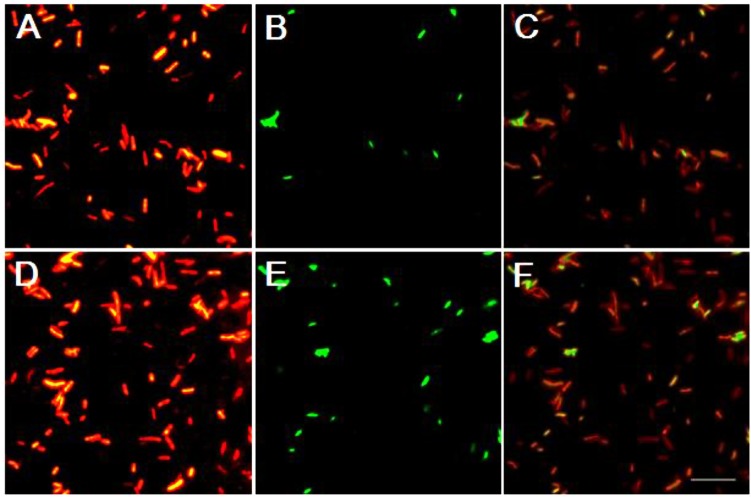
FISH analysis of the two dominant bacterial phylotypes of *Epsilonproteobacteria* and *Bacteroidetes* in the intestines of CMC. Every two epifluorescence photomicrographs (A and B, D and E) show the same microscopic field of CMC intestinal sample double-hybridized with oligonucleotide probes specific for *Bacteria* (Eub338) (A, D) and for *Epsilonproteobacteria* (EPSY549) (B) or *Bacteroidetes* (CF319a) (E). (C) Merged photo of A and B. (F) Merged photo of D and E. Scale bar = 10.0 μm.

To confirm the results obtained from the clone library, the intestinal bacteria were hybridized with fluorescence-labeled oligonucleotide probes targeting two dominant phylotypes of *Bacteroidetes* and ε-*Proteobacteria* (*Arcobacter*) found in the clone library. The results showed that all bacterial cells were able to hybridize with probe Eub338 (Fig 2), but not with probe antiEub338 (reverse complement probe Eub338), which indicated that the sample preparation and hybridization system were normal. More bacterial cells were detected with probe CF319a (targeting *Bacteroidetes*). In contrast, probe EPSY549 (targeting ε-*Proteobacteria*) only hybridized to a few bacterial cells (Fig 2).

### Bacterial morphology and colonization in CMC intestines

**Fig 3:**
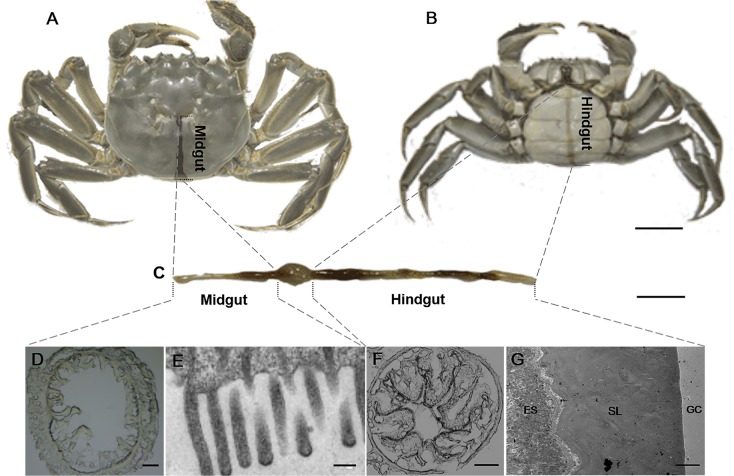
The location (A, B) and structure (C-G) of CMC gut. The cross-sections of CMC intestines were analyzed with light microscope (D, F) and with transmission electron microscope (E, G). ES represents endothelial cell surface, SL represents secretary layer, and GC represents gut cavity. Scale bar = 1.0 cm (A, B), 2.0 cm (C), 100 μm (D, F), 0.25 μm (E), and 5.0 μm (G).

**Fig 4:**
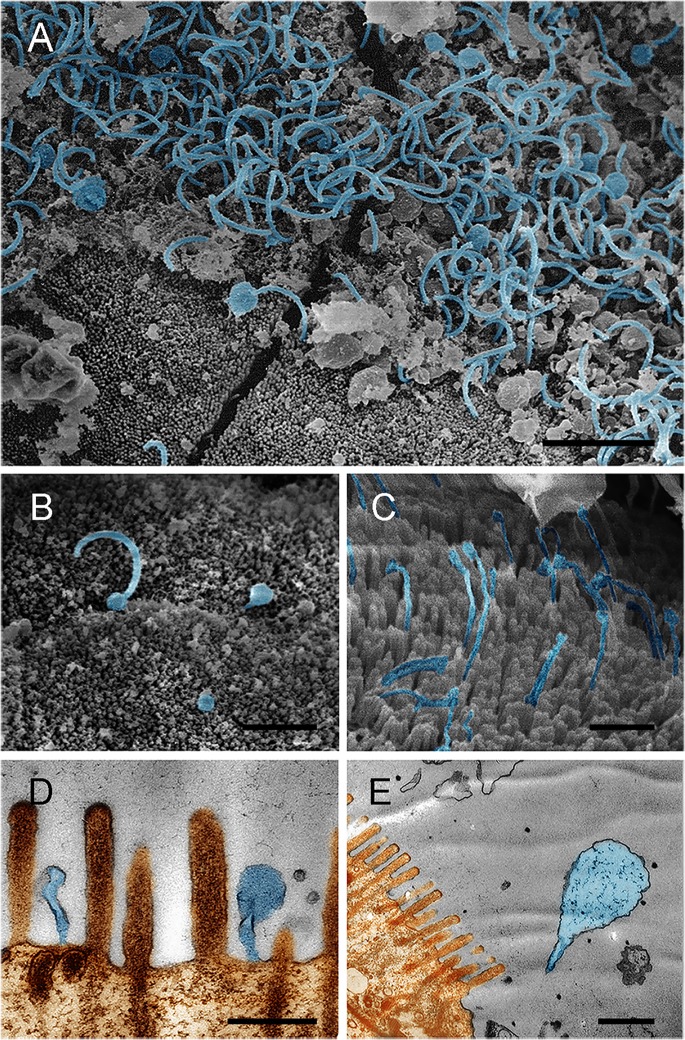
The bacterial morphology and association with the microvillus brush border in the midgut of CMC. Scanning electron microscopy (A, B, C) and transmission electron microscopy (D, E), show spherical and filamentous bacteria in close association with the microvilli. A bud-like cell protrusion at the end of the filaments was observed (A, B). Bacteria inserted into the space between the microvilli using a stalk-like cell appendage (D, E). Brown represents the epithelium and microvilli, and light blue indicates the bacteria. Scale bar = 2.0 μm (A), 4 μm (B), 1.0 μm (C), 0.5 μm (D and E).

To provide insight into the morphology and colonization of gut bacteria in CMC intestines, electron microscopy was performed. The intestine of adult crab is composed of two parts, midgut and hindgut (Fig 3). The midgut, approximately one-third of the total gut length, is located close to the dorsal shell of the crab (Fig 3A) and is where central digestion and absorption occur. Numerous filamentous and spherical bacteria were observed in the midgut. In addition, these bacteria were closely associated with the microvillus brush border (Fig 4A and 4B) and were inserted into the space between the microvilli (Fig 4C) or by a stalk-like cell appendage (Fig 4D and 4E). Moreover, a bud-like protrusion at the end of the cell was observed in these filamentous bacteria (Fig 4A, 4B and 4C). In contrast, few bacteria were observed in the enteric cavity.

**Fig 5:**
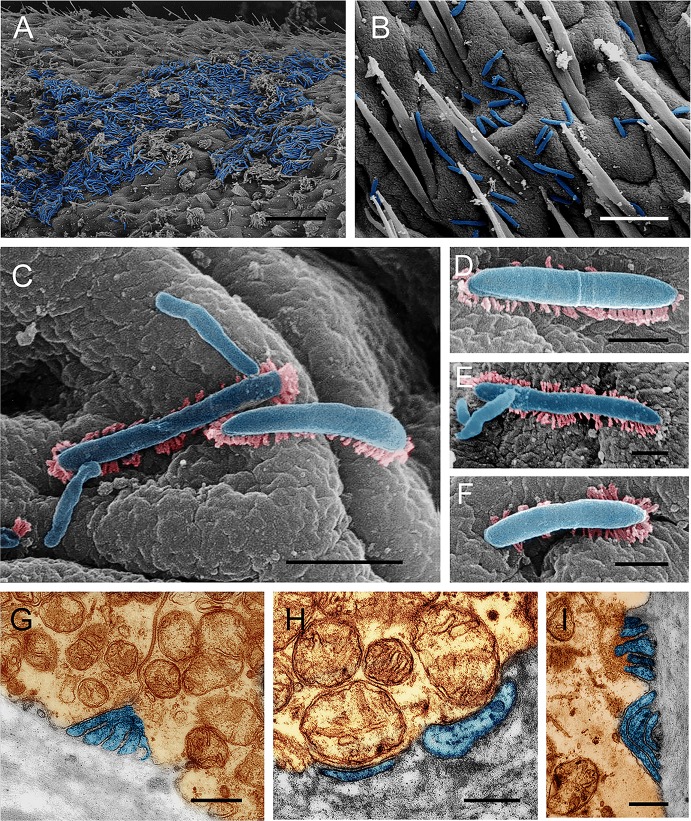
The bacterial morphology and association with the epithelium surfaces in the hindgut of CMC. Scanning electron microscopy (A-F) and transmission electron microscopy (G-I), show that rod-shaped bacteria sticking to the cell surface directly or by pili-like structures (C-F), bacteria invaginating the epithelium (G, I). Brown represents the epithelium, light blue indicates the bacteria, and pink represents the pili-like structure. Scale bar = 15.0 μm (A), 5.0 μm (B, G, I) and 1.0 μm (C), and 0.4 μm (D, E, F, H).

In the hindgut, the most abundant bacteria were in rod shape. These bacteria, attaching to the secretion layer of epithelium surface (Fig 3G), appeared to take advantage of fimbriae (pili)-like projections for adherence (Fig 5C, 5D, 5E and 5F). The bacteria located under the secretion layer were tightly attached to the epithelial surface (Fig 5G, 5H and 5I) and often led to the invagination of the epithelium surface (Fig 5G and 5I). Similar to the midgut, few bacterial cells were found in the enteric lumen of the hindgut.

### DGGE analysis of bacterial community in the midgut and hindgut

**Fig 6:**
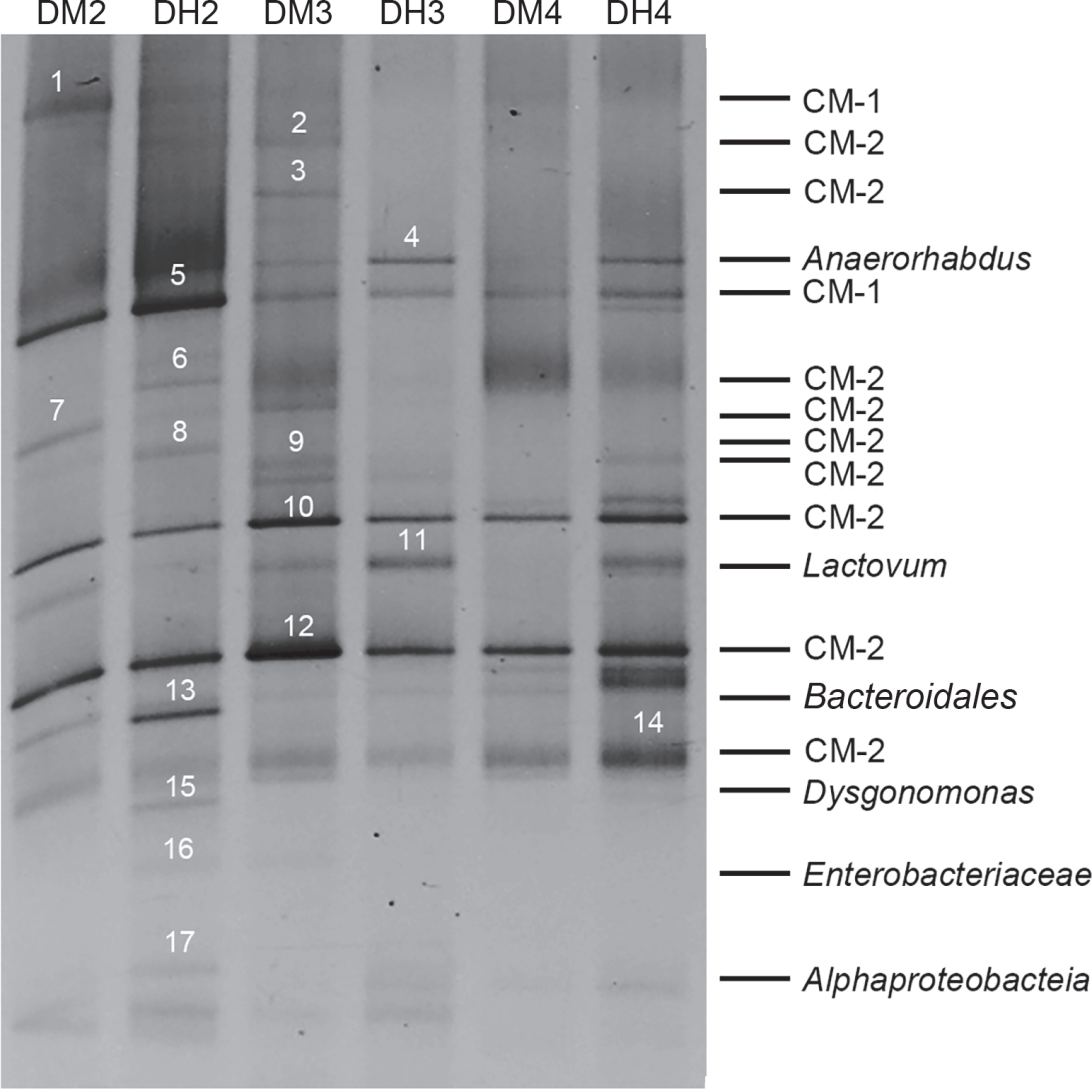
DGGE analysis of the intestinal bacterial community of CMC. The seventeen bands excised for sequencing were marked with numbers. DMx and DHx indicate midgut and hindgut, respectively. The samples were obtained from the same crab when x is of the same number. CM-1 represents CMC *Mollicutes* group 1 and CM-2 represents CMC *Mollicutes* group 2.

**Fig 7:**
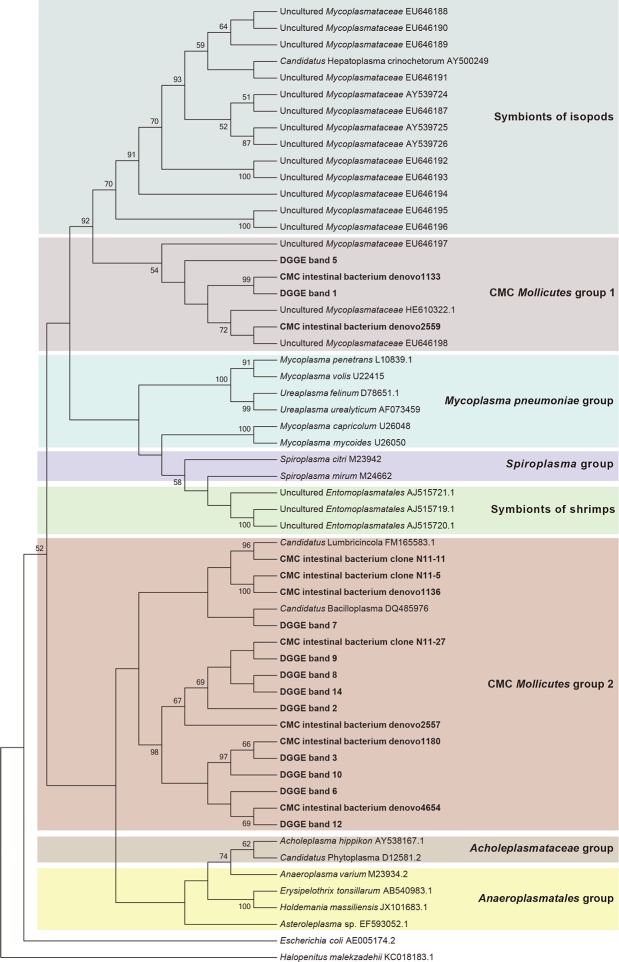
Phylogenetic affiliations of the *Mollicutes* 16S rRNA genes obtained from crab gut. Sequences derived from the present study are shown in bold. *Halopenitus malekzadehii* was used as an out-group to root the tree.

To understand the similarities and differences of bacterial community between the midgut and hindgut, DGGE analysis of the V1-V3 region of 16S rRNA gene was performed (Fig 6). A total of 17 bands were excised and cloned (Fig 6). Fifty clones were successfully sequenced. The inserts ranged from 490 to 527 bp in size. Based on RDP classifier and phylogenetic analysis, the cloned sequences from the 17 bands were categorized into four phyla, *Tenericutes*, *Bacteroidetes*, *Firmicutes* and *Proteobacteria* (Table 3). Among them, 11 bands (1, 2, 3, 5, 6, 7, 8, 9, 10, 12, 14) represented sequences from *Tenericutes*, three bands (4, 13, 15) belonged to *Bacteroidetes*, two bands (16, 17) were identified as *Proteobacteria*, and one band (11) was classified as *Firmicutes*. All CMC *Tenericutes* were phylogenetically clustered into two groups. The CMC *Mollicutes* group 2 (bands 2, 3, 6, 7, 8, 9, 10, 12, 14) shared >97% identity with each other, indicating they belonged to closely related species. In addition, they also shared > 97% similarity with a specific clone N11-27 based on local BLAST analysis of the DGGE sequences against the culture-independent 16S rRNA clone library sequences (128 nearly full length sequences), and they were affiliated to *Candidatus* Bacilloplasma and *Candidatus* Lumbricincola (Fig 7). The CMC *Mollicutes* group 1 (bands 1 and 5) shared >95% identity with the uncultured *Mycoplasmataceae* (HE610322.1), which was detected in mud crab (*Scylla paramamosain*). The CMC *Bacteroidetes* were composed of *Anaerorhabdus*, *Dysgonomonas* and one unclassified species. The CMC *Proteobacteria* consisted of *Citrobacter* and the unclassified *Proteobacteria*, and the CMC *Firmicutes* were related to *Lactovum*. The two CMC *Mollicutes* groups and the unclassified *Bacteroidales* (band 13) were detected in both the midgut and hindgut of all three samples (Fig 6).

**Table 3:** RDP Classifier (Confidence threshold: 95%) results of bacterial community in the CMC intestines based on DGGE.

Band no.	Gut^1^	Bacterial affiliation
1, 2, 3, 5, 6, 7, 8, 9, 10, 12, 14	M, H	Unclassified Bacteria
4	M, H	*Bacteroidetes*, *Bacteroidia*, *Bacteroidales*, *Bacteroidaceae*, *Anaerorhabdus*
11	M, H	*Firmicutes*, *Bacilli*, *Lactobacillales*, *Streptococcaceae*, *Lactovum*
13	M, H	*Bacteroidetes*, *Bacteroidia*, *Bacteroidales*
15	H	*Bacteroidetes*, *Bacteroidia*, *Bacteroidales*, *Porphyromonadaceae*, *Dysgonomonas*
16	M, H	*Proteobacteria*, *Gammaproteobacteria*, *Enterobacteriales*, *Enterobacteriaceae*, *Citrobacter*
17	H	*Proteobacteria*, *Alphaproteobacteria*

^1^ M, midgut; H, hindgut.

### Metagenomic analysis of bacterial community in the midgut and hindgut of CMCs

**Fig 8:**
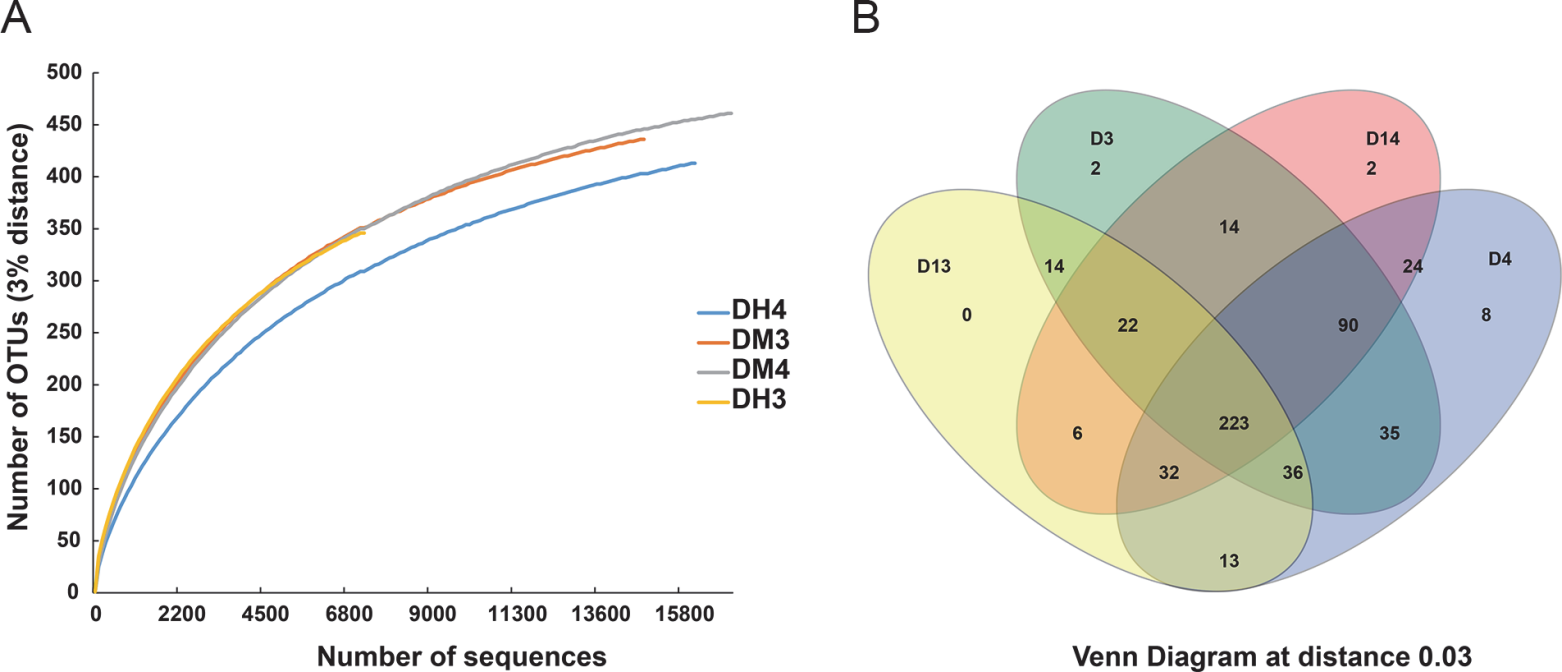
Rarefaction curves (A) and Venn diagram (B) of the bacterial 16S rRNA gene metagenomic data sets from the intestine of CMCs at 97% similarity. The rarefaction curves for all the samples reached the near plateau phase, representing good sampling depth. Most of the abundant species were common to all the samples. DMx and DHx indicate midgut and hindgut, respectively. The samples were obtained from the same crab when x is of the same number.

Metagenomic libraries were constructed in order to provide more information on the bacterial community in crab midgut and hindgut. A total of 61,105 reads were generated from four samples (67,555 raw reads) after substracting quality control, and 521 OTUs were assigned. OTU rarefaction curves became flatter with the increasing number of sequences (Fig 8A), which suggested that the sequencing cover the majority of bacterial species in the samples. OTU richness, coverage and diversity richness were calculated for each data set. Sample DH3 showed the least species richness compared to the other samples at the 97% similarity level (Table 4). Venn diagram at a distance of 0.3 was constructed to evaluate the distribution of OTUs among different samples. The results showed that 223 OTUs were shared by all samples, and the number of specific OTUs was less than 13 (Fig 8B).

**Table 4:** OTU richness, coverage and diversity richness index of the 16S rRNA gene obtained from metagenomic sequencing.

Sample ID	No. of raw sequences	No. of high quality sequences	No. of OTUs	Simpson	Chao1	Shannon	Good’s coverage
DM3	18,545	14,978	436	0.13	467.23	3.02	0.99
DH3	9,001	7,310	346	0.05	406.43	3.64	0.98
DM4	21,094	17,153	461	0.29	497.52	2.60	0.99
DH4	18,915	16,272	413	0.10	457.03	3.03	0.99

**Fig 9:**
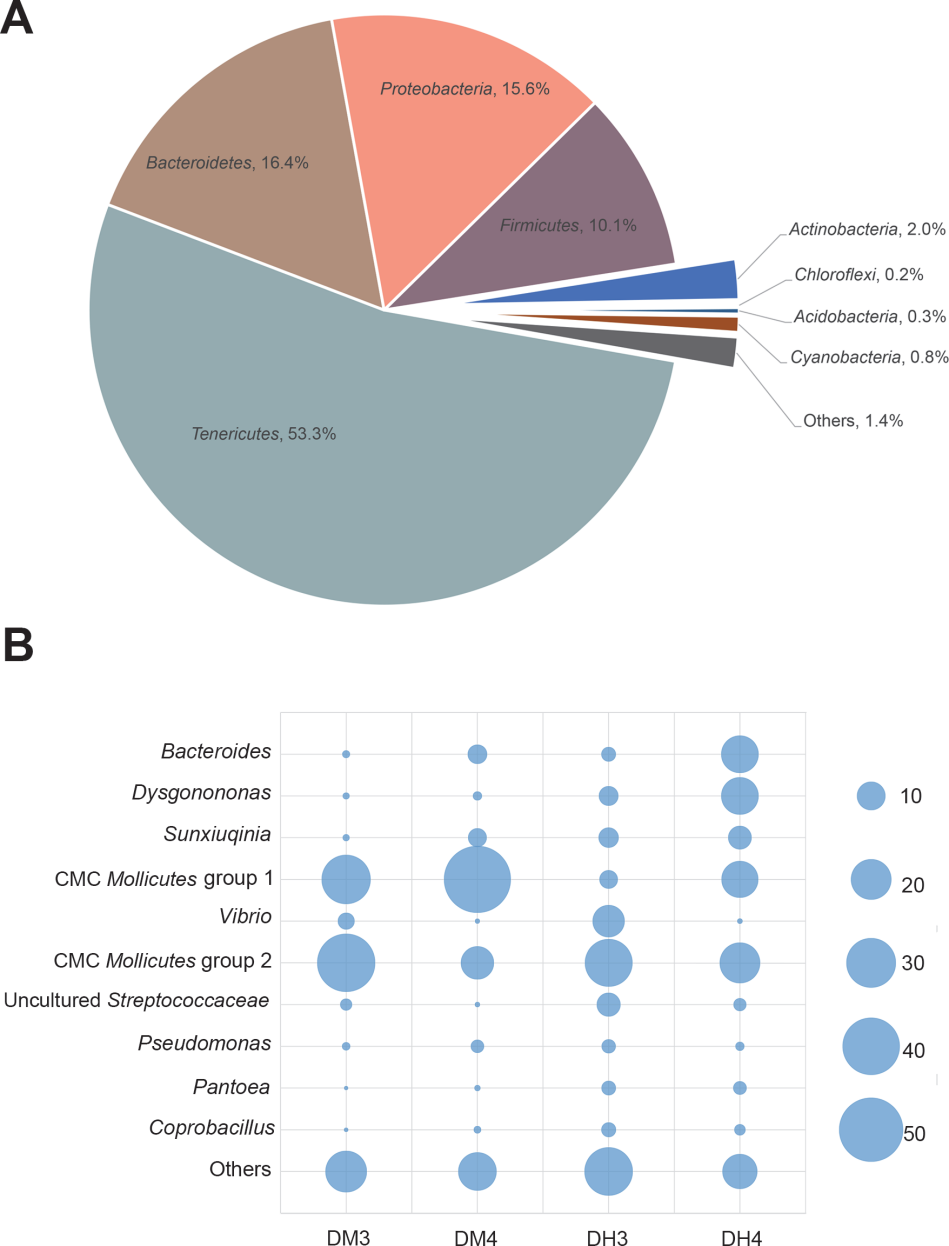
Relative abundance of bacterial species on phylum (A) and dominant OTUs level (B) in the 16S rRNA gene metagenomic data sets obtained from the midgut and hindgut of CMCs. The CMC Mollicutes group 1 and group 2 are classified based on phylogenetic analysis. The size of blue pie graph indicates the different abundance of OTUs.

Sequences were classified into eight phyla, *Tenericutes*, *Bacteroidetes*, *Proteobacteria*, *Firmicutes*, *Actinobacteria*, *Acidobacteria*, *Chloroflexi*, *Cyanobacteria*, and unclassified bacteria (Fig 9A). The more abundant phyla were *Tenericutes* (53.3%), *Bacteroidetes* (16.4%), *Proteobacteria* (15.6%) and *Firmicutes* (10.1%), which were similar to the results from the clone library and DGGE.

**Fig 10:**
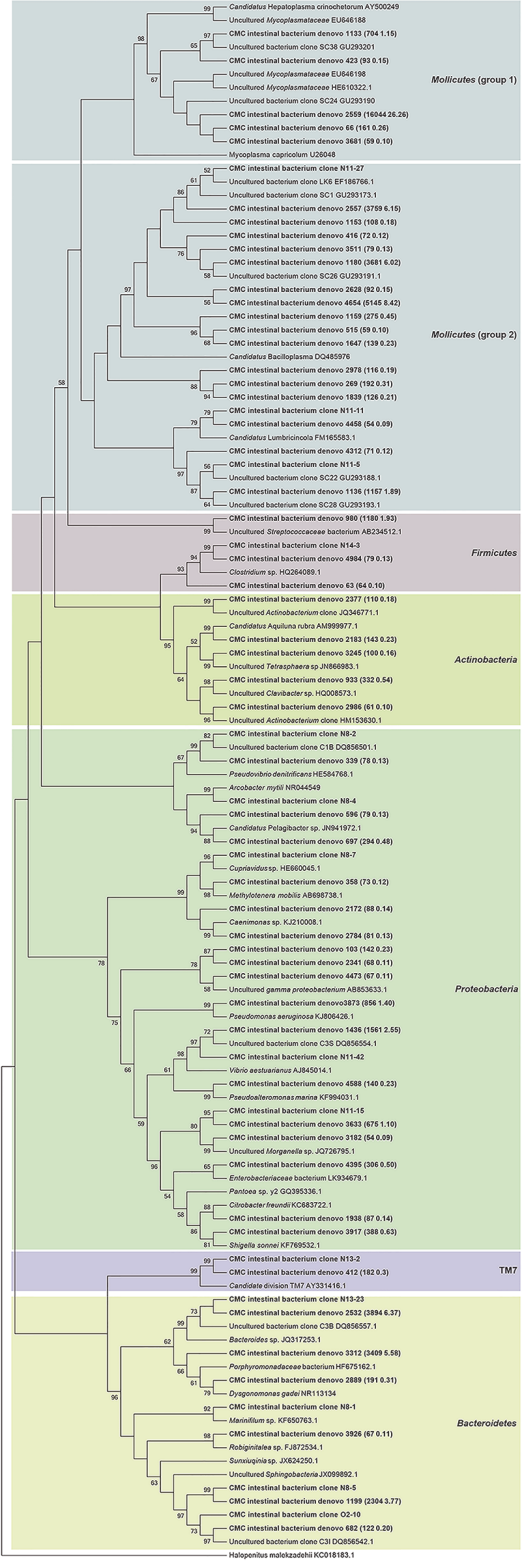
Phylogenetic affiliations of dominant OTUs from 16S rRNA metagenomic analysis of CMC intestines. Phylogenetic affiliations of dominant OTUs that contain more than 50 reads in the bacterial 16S rRNA gene metagenomic data sets obtained from crab intestines. Sequences derived from the present study are shown in bold. The background colors indicate the bacterial phylotypes of different phyla.

At the genus level, 29.8% of the sequences were classified at 97% identity against the SILVA database (SSU). *Bacteroides*, accounting for 6.37% of sequences, was the most abundant genus represented, while most other genera were only represented by less than 1% of all sequences. In addition, 70.2% of sequences were unclassifiable at the genus level. Unique representative sequences from each dominant OTUs that contained more than 50 reads were compared to GenBank non-redundant database. The top hits were included, along with the representative sequences, in the phylogenetic analysis. Sixteen OTUs (24.8% of total OTUs), which were similar to the dominant *Mollicutes* OTU N11-27 in the clone library, were closely related to the CMC *Mollicutes* group 2. In addition, five OTUs, which represented 28.0% of all sequences, were related to the CMC *Mollicutes* group 1 (Fig 10). For the *Bacteroidetes*, three groups were closely related to *Bacteroides* (7.0%), *Dysgonomonas* (6.1%) and *Sunxiuqinia* (4.2%), respectively. For the *Proteobacteria*, 2.9% of the sequences were related to *Vibrio*, and 1.4% of the sequences were related to *Pseudomonas* (Fig 10).

All the dominant OTUs were found in both the midgut and hindgut; however, their abundances in the different intestinal regions were different (Fig 9B). The number of CMC *Mollicutes* group 1 in the midgut was higher than that in the hindgut. Conversely, *Bacteroides* and *Dysgonomonas* were more abundant in the hindgut. Other phylotypes, such as the CMC *Mollicutes* group 2, uncultured *Streptococcaceae*, *Sunxiuqinia*, *Pseudomonas*, *Pantoea* and *Coprobacillus* showed no apparent differences in abundance between the midgut and hindgut (Fig 9B).

## Discussion

This investigation is a systematic study that examined the intestinal bacterial community in CMCs farmed in Lake Tai, China, through employing various experimental methods. The results have uncovered several intriguing aspects of crab intestinal bacteria that have not been previously reported.

### Potential indigenous gut bacteria

In the present study, *Bacteroidetes*, *Tenericutes*, *Firmicutes*, and *Proteobacteria* represent the four dominant phylotypes colonizing the intestine of farmed CMCs from Lake Tai (Table 5). They may represent the indigenous intestinal bacterial community in CMC populations.

**Table 5:** Phylotypes of the core bacterial community in the CMC intestines.

Representative OTU	RDP classifier (Bootstrap value, %)	BLAST best hits		Percentage (%)		
		UB^1^ source (Identity, %)	Known species (Identity, %)	Clone library^2^	Metagenomic^3^	Li et. al.^4^
N13-23	*Bacteroides* (100)	Intestinal tract of CMC (99)	*Bacteroides* sp. (99)	0.8	7.0	12.2
N8-5	*Meniscus* (100)	Intestinal tract of CMC (99)	*Sunxiuqinia* sp. (91)	19.3	4.2	16.0
N8-1	*Marinifilum* (100)	Intestinal tract of CMC (99)	*Marinifilum* sp. (94)	13.8	0	10.3
N13-1	*Lachnospiraceae* (98)	Intestinal tract of CMC (99)	*Clostridium colinum* (88)	2.3	0	2.8
N14-3	*Clostridium XlVb* (95)	Gut of *Polyphagous lepidopteran* larvae (91)	*Clostridium colinum* (90)	5.4	0.2	0
N11-27	*Mollicutes* ^*5*^	Stomach of yellow catfish (97)	*Candidatus* Bacilloplasma (86)	7.7	16.6	0
N11-5	*Mollicutes* ^*5*^	Stomach of yellow catfish (99)	*Abiotrophia defectiva* (84)	11.5	2.1	0
N11-15	*Enterobacteriaceae* (100)	Endosymbiont of *Onthophagus taurus* (97)	*Pantoea* sp. *y2* (97)	8.5	1.2	0
N15-67	*Vibrio* (100)	Intestinal tract of CMC (99)	*Vibrio aestuarianus* (96)	2.3	2.9	0.3
N8-2	*Alphaproteobacteria* (99)	Intestinal tract of CMC (99)	*Pseudovibrio denitrificans* (88)	5.4	0.2	9.7
De2559	Bacteria (99)	Stomach of yellow catfish (96)	Uncultured *Mycoplasmataceae* (95)	0	28.8	0
De3312	*Bacteroidetes* (100)	Intestinal tract of CMC (100)	*Dysgonomonas capnocytophagoides* (87)	0	6.1	1.3

^1^ UB, uncultured bacteria;

^2^ Percentage among the 128 cloned sequences (>97 identity);

^3^ Percentage among the 5,5713 metagenomic reads (>97 identity);

^4^ Percentage among 319 cloned sequences (Li et. al. (2007));

^5^ OTUs were classified to *Firmcutes* based on RDP. However, phylogenetic analysis showed they belonged to *Mollicutes*.

The CMC *Bacteroidetes* appear to be pervasive within CMC population and are maintained persistently through time. This group comprises three phylotypes, namely, two uncultured *Bacteroidetes* and the *Bacteroides*. They share >99% sequence similarity with the cloned 16S rRNA genes of the *Bacteroidetes* commonly found in the CMCs collected from the Yangtze River estuary and Chongming Islands, China [9]. Despite differences in water environments, diets, and the temporal and spatial separation [9], all the CMC guts analyzed contain similar *Bacteroidetes*, which suggest that the *Bacteroidetes* are closely associated with their crab hosts. This speculation is further supported by FISH analysis of nine crabs. The aforementioned *Bacteroidetes* were detected in the homogenate suspension from all of the crab intestines. Accordingly, the CMC *Bacteroidetes* might be the indigenous bacteria specific to the crab intestine and comprise the core microbiota in the gut of CMCs.

Based on the 16S rRNA sequence similarity assay, *Marinifilum fragile* (*Bacteroidetes*) isolated from tidal flat sediment in Korea [21] is the closest relative to the *Marinifilum* found in crab intestines. In addition, *M*. *fragile* is a filamentous bacterium [21], which is morphologically very similar to the bacteria found in the crab intestine. Similar to *M*. *fragile*, the crab gut filamentous bacteria possess also a bud-like protrusion at the end of their cells. Accordingly, both sequence and morphology analyses indicate that a *M*. *fragile* related species may reside in the crab midgut. To further confirm this speculation, FISH specific for the *Bacteroidetes* was performed by using the thin sections of crab gut. Unfortunately, the thin sections revealed strong autofluoresence and nonspecific binding of the supposedly *Bacteroidetes*-specific probe (data not shown). Although the hybridization procedures were optimized, it failed to distinguish the positive signals from the strong background signals of the thin sections.

As shown in the phylogenetic tree (Fig.1), the *M*. *fragile* related phylotype was clustered with the functionally unknown endosymbiont of the bone-eating worm *Osedax mucofloris* from shallow whale-falls in the North Atlantic [22]. It was assumed that the *Osedax* endosymbionts were taken up from the marine environment by *O*. *mucofloris* [22]. Given that *M*. *fragile* is a marine bacterium, it is reasonable to speculate that the CMC gut *M*. *fragile*-like bacteria originated in the sea. Since *M*. *fragile* is the only species in the genus *Marinifilum* and very little is known about its ecological roles, it is rather impossible to predict its associations with CMCs.

Thus far, the *Marinifilum* bacteria were only observed in the gut of farmed CMCs but not in wild ones. Moreover, several less abundant *Bacteroidetes* phylotypes, like *Flavobacterium*, are present in the crabs from Chongming Islands but absent in those from Lake Tai. Whether these discrepancies result from diet and/or habitat differences is still unclear and warrants further study.

*Bacteroides* species are commonly detected in the gut of animals including shrimps, fish and humans [23]. They are thought to be indigenous to the host and maintain a complex and generally beneficial relationship with the host [23]. While, thus far, close relatives of the CMC *Bacteroides* species have not been observed in other animals and environments. Therefore, it is reasonable to suggest that they may have a specific relationship with CMCs. The abundance of *Bacteroides* is higher in the hindgut than in the midgut and appears to vary between individuals.

The 16S rRNA clone library, DGGE and metagenomics assays indicate that the CMC guts contain bacteria that are related to *Mollicutes*. Surprisingly, all the *Mollicutes* phylotypes detected in the CMCs are closely related to those found in the stomach of yellow catfish collected from Niushan Lake, Hubei province, China [24]. While the examined catfish was young and weighted approximately 80 g, the crabs used in this study were adults and weighted more than 150 g. Therefore, the similar *Mollicutes* phylotypes found in catfish stomach are unlikely to have originated from the consumption of adult CMCs by the catfish. However, such *Mollicutes* might be present in the preys of catfish, like shrimps, isopods, or others.

The CMC *Mollicutes* groups 1 and 2 may be the resident symbionts colonizing the CMC intestines. The CMC *Mollicutes* group 2 is closely related to *Candidatus* Bacilloplasma and *Candidatus* Lumbricincola [25, 26], and appears to be present in both the midgut and hindgut without significant difference in abundance. However, the CMC *Mollicutes* group 1 was found mostly in the midgut of CMCs (Fig 9B); they share more than 95% sequence identity with the uncultured *Mycoplasmataceae* detected in mud crab (*Scylla paramamosain*) and share over 96% sequence identity with the uncultured *Mycoplasmataceae* symbionts in the midgut of marine isopod *Ligia oceanica* (Crustacea: Isopoda) [27]. This group of uncultured *Mycoplasmataceae* is affiliated with “*Candidatus* Hepatoplasma crinochetorum” which is a hepatopancreatic symbiont of terrestrial isopods [11]. Since crabs and isopods both belong to the *Malacostraca* species, the CMC *Mollicutes* group 1 may derive from a common ancient ancestor shared by the isopod mycoplasmas. The contributions of these bacteria to their host remain unclear. However, isopods that harbor *Candidatus* Hepatoplasma have been shown to have a higher chance of survival under nutritional stress (low-quality food) [27]. It is worth noting that the CMC *Mollicutes* group 1 found in this study is absent in the intestines of CMCs sampled from the Yangtze River estuary and the Chongming Islands [9]. Therefore, the *Mollicutes* appear not to be indispensable to the crab host.

The unclassified *Alphaproteobacteria*, which shares 99% identity with the 16S rRNA gene sequence of the phylotype N8-2, is only found in the hindgut of crabs (Fig 6). These bacteria were also found in CMCs collected from the Yangtze River estuary and Chongming Islands [9]. However, they share less than 90% similarity with other sequences in GenBank database, which suggests that this unclassified *Alphaproteobacteria* may represent a CMC-specific species that mainly reside in the hindgut of CMCs.

Based on results from the clone library of 16S rRNA genes, the *Arcobacter* phylotype (ε-*Proteobacteria*), the most abundant phylotype in CMCs from Lake Tai, shares 98.4% sequence similarity with the *Arcobacter mytili* strains isolated from mussels [28]. Several *Arcobacter* species are human enteropathogens, for examples, *A*. *butzleri* and *A*. *cryerophilus* [29]. Members of *Arcobacter* are also detected in the intestinal tracts and fecal samples of different farmed animals, but few of them cause disease [29]. Given that the crabs used in this study were healthy and that their intestines showed no obvious signs of diseases, the speculation that the *Arcobacter* phylotypes found in these intestines are pathogenic seems unlikely. However, it is possible that these *Arcobacter* bacteria are pathogenic in human. The *Arcobacter* phylotypes were detected in crabs in this study but were absent in other CMC populations [9]. Therefore, it is unlikely that these *Arcobacter* phylotypes are residents of CMC intestines. They may be ingested by crabs living in Lake Tai.

Three *Firmicutes* phylotypes were detected in the gut of CMCs in this study. One of them belongs to an unclassified lineage. This unclassified phylotype (OTU representative N13-1) shared >99% sequence similarity only to the *Firmicutes* found in intestines of CMCs collected from the Yangtze River estuary and the Chongming Islands [9]. It is reasonable to speculate that these gut bacteria, shared by different crab populations but not in other aqua-cultured animals such as shrimp and fish, are dependent on host specificity [4, 5, 30–32] and may represent some of the CMC-specific bacterial species that comprise the core microbiota in CMC intestines.

### The potential role of observed stalk- and pili-like structures

Both transmission electron microscopy and scanning electron microscopy reveal an intimate association between the intestinal bacteria and the epithelial surfaces of both the midgut and the hindgut of CMCs. In the midgut, the filamentous and spherical bacteria insert into the space between microvilli using a stalk-like cell appendage. Interestingly, the midgut symbiotic bacteria, *Candidatus* Hepatincola porcellionum and *Candidatus* hepatoplasma crinochetorum of isopod *Porcellio scaber* (Crustacea: Isopoda), also interact with the microvillus brush border of the epithelium by means of a similar cell protrusion structures of unknown functions [11, 33]. In addition, sequence analysis confirms that the CMC *Mollicutes* are closely related to the isopod *Mycoplasmataceae* symbionts, which contain *Candidatus* hepatoplasma crinochetorum. If the bacteria indeed depend on the appendage structure for specific colonization of CMC midgut, the similar appendages observed in both CMCs and isopods likely resulted from convergent evolution. To our knowledge, such structure is rarely observed in the midgut bacteria of other animals, which might employ alternative colonization strategies. For example, a nipple-like appendage in some indigenous intestinal bacteria has been documented to insert into epithelial cell without physically penetrating it [34, 35].

Unlike the bacteria in the midgut, the rod-shaped bacteria in the hindgut appear to possess a pili-like structure. This structure could be beneficial for the bacteria and enable them to attach tightly to the epithelial surfaces without microvilli and to avoid being expelled by the hindgut lumen. In 2007, Kostanjsek and co-authors reported that the rod-shaped bacteria *Candidatus* Bacilloplasma colonize the cuticular surface of the terrestrial isopod *Porcellio scaber* (Crustacea: Isopoda) [25]. Interestingly, these bacteria attach to the cuticular spines by a spherical structure at the tapered end of the bacterial cell, which results in the formation of a rosette-like structure [25]. Both the pili-like and the rosette-like structures seem to contribute to the attachment of gut bacteria to the intestinal epithelium. Although the CMC *Mollicutes* group 2 is closely related to the aforementioned *Candidatus* Bacilloplasma, whether they are the pili-formed cells identified in the crab intestines is still in question. In 1976, in their effort to understand the mechanism of colonization of the intestinal epithelium of rabbits by *Vibrio cholerae*, Nelson et al. observed *V*. *cholerae* with pili-like structure [36]. They augured that, due to the rarity of the observation, this structure may either be an artifact from the sample preparation or a pili structure involved in adhesion. In contrast, in this study, many rod-shaped bacteria attached to the epithelial surface have the pili-like structures. In addition, we speculate that the pili-like structure reported by Nelson et al. might be from some resident bacteria that present at low density in rabbit gut, but are not part of the experimentally injected *V*. *cholerae*. The unusual pili-like and stalk-like structures of gut bacteria probably have some functional contributions, such as improved attachment or survive in the gut environment, which suggests that the bacteria with these structures are probably crab intestinal autochthonous.

In conclusion, this study reveals a novel and morphologically unusual bacterial community in the intestines of CMCs farmed in Lake Tai, China. They comprise four dominant phylotypes of *Tenericutes*, *Bacteroidetes*, *Firmicutes*, and *Proteobacteria*, which may represent the indigenous intestinal bacterial community in CMC populations. To further confirm this speculation, more crab samples from distinct farming conditions as well as at different growth stages need to be subject to analysis and comparison.
